# The NMDA antagonist memantine affects training induced motor cortex plasticity – a study using transcranial magnetic stimulation [ISRCTN65784760]

**DOI:** 10.1186/1471-2202-6-35

**Published:** 2005-05-12

**Authors:** Peter Schwenkreis, Katja Witscher, Burkhard Pleger, Jean-Pierre Malin, Martin Tegenthoff

**Affiliations:** 1 Department of Neurology, Ruhr-University Bochum, BG-Kliniken Bergmannsheil, Buerkle-de-la-Camp-Platz 1, D-44789 Bochum, Germany

## Abstract

**Background:**

Training of a repetitive synchronised movement of two limb muscles leads to short-term plastic changes in the primary motor cortex, which can be assessed by transcranial magnetic stimulation (TMS) mapping. We used this paradigm to study the effect of memantine, a NDMA antagonist, on short-term motor cortex plasticity in 20 healthy human subjects, and we were especially interested in possible differential effects of different treatment regimens. In a randomised double-blinded cross over study design we therefore administered placebo or memantine either as a single dosage or as an ascending dosage over 8 days. Before and after one hour of motor training, which consisted of a repetitive co-contraction of the abductor pollicis brevis (APB) and the deltoid muscle, we assessed the motor output map of the APB muscle by TMS under the different conditions.

**Results:**

We found a significant medial shift of the APB motor output map after training in the placebo condition, indicating training-induced short-term plastic changes in the motor cortex. A single dosage of memantine had no significant effect on this training-induced plasticity, whereas memantine administered in an ascending dosage over 8 days was able to block the cortical effect of the motor training. The memantine serum levels after 8 days were markedly higher than the serum levels after a single dosage of memantine, but there was no individual correlation between the shift of the motor output map and the memantine serum level. Besides, repeated administration of a low memantine dosage also led to an effective blockade of training-induced cortical plasticity in spite of serum levels comparable to those reached after single dose administration, suggesting that the repeated administration was more important for the blocking effect than the memantine serum levels.

**Conclusion:**

We conclude that the NMDA-antagonist memantine is able to block training-induced motor cortex plasticity when administered over 8 days, but not after administration of a single dose. This differential effect might be mainly due to the prolonged action of memantine at the NMDA receptor. These findings must be considered if clinical studies are designed, which aim at evaluating the potency of memantine to prevent "maladaptive" plasticity, e.g. after limb amputation.

## Background

Determination of motor output maps by transcranial magnetic stimulation (TMS mapping) proved to be a useful tool allowing the study of the cortical representation of various muscles [1-5], and showing a high map stability and reproducibility [6-8]. Serial TMS mappings can be used to assess short-term plastic changes of the motor cortex induced by the repetitive performance of a motor task consisting of a synchronised movement of two limb muscles. This was demonstrated by a shift of the centre of gravity (COG) of the motor output map derived from a small hand muscle towards the representation of the co-contracted shoulder [9] or leg muscle [10]. Hence this model was used to study the effect of different central acting drugs on short-term motor cortex plasticity: The GABA_A _agonist lorazepam and the N-methyl-D-aspartate (NMDA)-antagonist amantadine were found to block such cortical plastic changes [11], whereas the indirect dopaminergic and adrenergic agonist amphetamine and the serotonin reuptake inhibitor fluoxetine enhanced training induced cortical plasticity [12,13]. Similar effects of GABA_A _agonists, NMDA antagonists and amphetamine were reported in series of studies using different paradigms to study training-induced motor cortex plasticity by TMS [14-16], and coactivation-induced plasticity in the primary somatosensory cortex [17-19]. These results support the view that short-term plastic changes in the motor cortex are based on functional changes of synaptic activity, requiring removal of local (presumably GABA_A _mediated) inhibition, as well as long-term potentiation (LTP)-like changes which are mediated through NMDA receptor activation [20-22].

Motor cortex plasticity, which occurs not only after motor training, but also after peripheral or central lesions [23], might not necessarily be adaptive or beneficial: Possible maladaptive consequences such as phantom limb pain after limb amputation are also discussed to be related to such cortical plastic changes [24]. Hence it could be of therapeutical interest to prevent cortical plasticity, e.g. by using drugs such as memantine, which is a non-competitive antagonist of glutamate and other excitatory amino acids at the MK-801-binding site of the NMDA receptor [25-27].

Here, we used the previously introduced muscle co-contraction paradigm (co-contraction of the deltoid and of the abductor pollicis brevis (APB) muscle) combined with TMS mapping of the APB muscle before and after training in order to evaluate the effect of memantine on short-term motor cortex plasticity in healthy subjects, and on motor performance. Previous studies generally used single dose administration of different drugs like NMDA antagonists or GABA agonists in order to pharmacologically modulate cortical plasticity [15,28,29]. Here, we were particularly interested in possible differential effects of different treatment regimens of memantine, and therefore administered placebo or memantine either as a single dosage or as an ascending dosage over 8 days to healthy humans in a randomised double-blinded cross over study design.

## Results

### Training effect on mapping parameters and map reproducibility

Looking at the placebo sessions of all 15 subjects participating in the placebo-controlled experiments, we found a significant medial shift of the amplitude-weighted centre of gravity (COG) of the motor output map of the APB muscle (y coordinate before training -5.12 ± 0.64 cm mean ± SD, after training -5.02 ± 0.58 cm, paired t-test, p = 0.022) after motor training. This COG shift was accompanied by a significant lowering of the motor threshold (MT before training 37.4 ± 6.0%, after training 36.7 ± 5.5%, p = 0.019). Other mapping parameters like the x coordinate of the COG, the area of the map, the number of hotspots of the map, the mean MEP amplitude and the sum of amplitudes (SOA) of the map did not change significantly during training.

Comparing the TMS mappings before motor training in the individual subjects between memantine and placebo sessions, there was no significant difference in one of the mapping parameters, indicating a high reproducibility of the mapping procedure.

### Memantine effect (single dosage experiment; Table 1)

**Figure 1:**
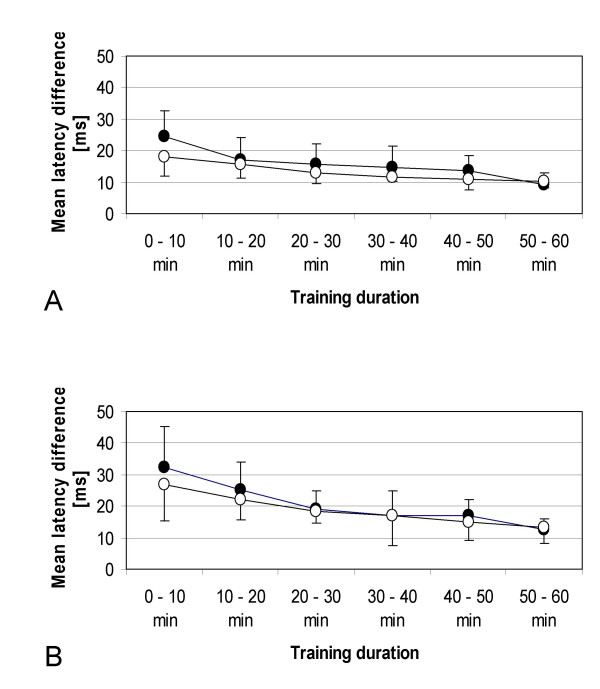
**Motor performance**. Comparison of motor performance with placebo (filled circles) and with memantine (open circles). Shown are the results of the single dosage (A) and ascending dosage (B) experiment. The mean latency difference between the onset of muscle contractions (abductor pollicis brevis and deltoid muscle) is shown at different motor training durations.

In the eight subjects who participated in this experiment, we found a significant shortening of the latency differences between the onset of the APB and deltoid muscle contraction during the course of the motor training as revealed by ANOVA for repeated measurements (significant influence of the factor "training duration", F(3.14,43.95) = 17.729, p < 0.001; Fig. 1A). Memantine did not affect this training effect, as shown by the non-significant influence of the factor "drug" (F(1,14) = 1.876, p = 0.192), and by the non-significant interaction between both factors (F(3.14,43.95) = 1.646, p = 0.191).

**Figure 2:**
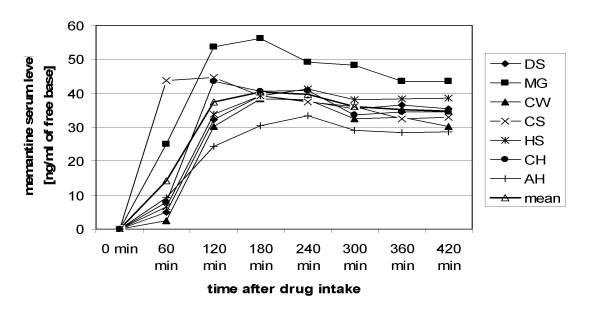
**Memantine serum levels (single dosage)**. Time course of the memantine serum level after administration of a single dosage. The serum levels of individual subjects, as well as the mean serum level at the different measurement points are shown.

Memantine serum levels, which were assessed in seven subjects, reached a plateau about 2 hours after drug intake and remained almost stable during the following 5 hours (Fig. 2). Among all subjects, the highest memantine serum level that was reached was 56.16 ng/ml of memantine free base (3 hours after drug intake). Mean memantine serum level 5 hours after drug intake (i.e., immediately after motor training, before starting the second mapping procedure) was 36.1 ± 6.1 ng/ml.

ANOVA revealed a significant lowering of MT after training (F(1,14) = 9.000, p = 0.010 for the factor "training"), but without significant influence of the factor "drug" (F(1,14) = 0.072, p = 0.793), and without significant interaction between both factors (F(1,14) = 0.111, p = 0.744). For other mapping parameters, no significant influence of the factors "training" or "drug", and no significant interaction between both factors was detected.

### Memantine effect (ascending dosage experiment; Table 1)

Similar to the results of the single dosage session, we found a significant shortening of the latency differences between the onset of the APB and deltoid muscle contraction during training as revealed by ANOVA for repeated measurements (significant influence of the factor "training duration", F(1.79,21.46) = 13.528, p < 0.001; Fig. 1B) in the seven subjects who participated in this experiment. Again, memantine did not affect this training effect, as shown by the non-significant influence of the factor "drug" (F(1,12) = 0.422, p = 0.528), and by the non-significant interaction between both factors (F(1.79,21.46) = 0.476, p = 0.607).

Mean serum level in the memantine session was 83.1 ± 17.4 ng/ml of memantine free base, with a maximum of 105.4 ng/ml in one subject.

**Figure 3:**
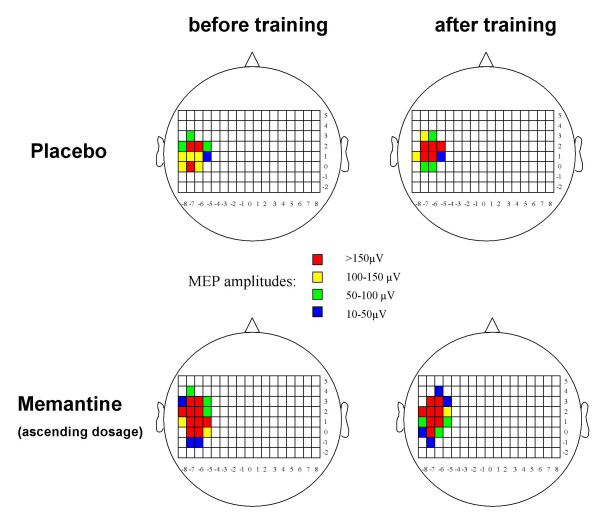
**TMS mapping (individual subject)**. Example of the TMS mapping results in one individual subject who participated in the ascending dosage experiment. Different colours represent the MEP amplitudes after stimulation at the different scalp positions. Note the medial shift of the motor output map of the abductor pollicis brevis muscle after training in the placebo condition, which is no longer present in the memantine condition.

Regarding the y coordinate of the COG, there was a medial shift of the y coordinate under placebo (+0.12 ± 0.18 cm), whereas under memantine a slight lateral shift was observed (-0.08 ± 0.17 cm). ANOVA revealed a significant interaction of the factors "training" and "drug" (F(1,12) = 4.789, p = 0.049), whereas the factors "training" (F(1,12) = 0.150, p = 0.705) and "drug" (F(1,12) = 0.000, p = 1.000) alone did not influence the y coordinate, indicating a significant difference between the shifts of the y coordinate under the different experimental conditions (Fig. 3). For other mapping parameters, no significant influence of the factors "training" or "drug", and no significant interaction between both factors was found.

In order to assess the effect of memantine alone on mapping parameters, without motor training, we additionally compared baseline TMS maps in the placebo and in the memantine condition using Student's paired t-test. For none of the mapping parameters, including the y-coordinate of the COG (-5.460 ± 0.692 with placebo, -5.359 ± 0.503 with memantine, p = 0.301), a significant difference could be observed between the baseline maps (Table 1), indicating that memantine alone had no effect on the mapping parameters.

### Influence of memantine serum levels

In order to determine whether the differential effect of memantine in the two experiments might be more related to the different serum levels or to the repeated dosage, we administered a low dosage of memantine (10 mg/d) over 8 days in 5 additional healthy subjects. Mean memantine serum level in this control experiment was 32.2 ± 5.4 ng/ml of memantine free base, with a maximum of 39.9 ng/ml in one subject, and therefore similar to the mean memantine level in the single dosage experiment. The training effect on motor performance was comparable to the other two experiments, with a significant shortening of the latency differences between the onset of the APB and deltoid muscle contraction during training as revealed by ANOVA for repeated measurements (significant influence of the factor "training duration", F(5,20) = 8.907, p < 0.001). However, for none of the mapping parameters, a significant pre-post difference could be observed after motor training (Table 1). Similarly to the ascending dosage experiment, the y co-ordinate of the COG showed a tendency rather to a lateral than to a medial shift, indicating an effective prevention of training-induced cortical plastic changes.

**Figure 4:**
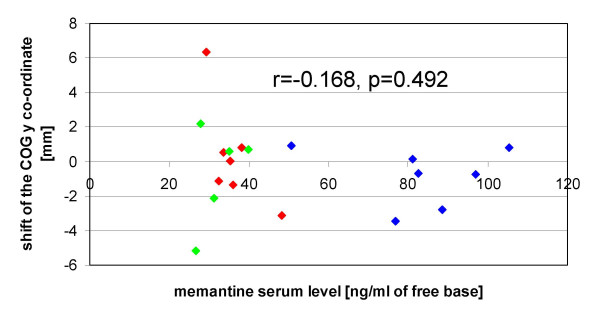
**Relationship between memantine serum levels and COG shift**. Memantine serum levels are plotted against the shift of the y coordinate of the COG after training. Positive values indicate a shift in a medial, negative values a shift in a lateral direction. Linear correlation analysis reveals no significant correlation. Subjects who participated in the single dosage experiment are red-labelled, subjects who participated in the ascending dosage experiment are blue-labelled, and subjects who participated in the control experiment (repeated low dosage) are green-labelled. Memantine serum levels of the single dosage experiment refer to the values obtained 5 hours after drug administration.

Looking separately at the different experiments, no significant individual correlation was found between memantine serum levels and the pre-post differences of one of the mapping parameters. With the data of the memantine conditions of all experiments pooled together, also no significant correlation with the memantine serum level was detected, neither for the pre-post difference of the y coordinate (Fig. 4), nor for one of the other mapping parameters.

### Relationship between motor performance and TMS mapping

For none of the pre-post differences of the mapping parameters, a significant correlation to the improvement of the motor performance could be observed.

## Discussion

The main finding of this study is that the repetitive performance of a motor task consisting of a synchronised contraction of the APB and deltoid muscle in the placebo condition leads to a directional shift of the COG of the motor output map of the APB muscle towards the more medial representation of the co-contracted muscle, and that this medial shift is blocked by memantine administered over a period of 8 days, but not by a single dose of memantine.

Our results confirm the results of previous studies, which demonstrated a similar directional shift by means of TMS mapping using the same motor training paradigm [11-13]. In addition, a training induced reduction of motor threshold was observed, which did not differ between placebo and memantine sessions. This lack of influence of memantine on motor threshold is in line with previous findings, suggesting that motor threshold mainly reflects the excitability of the neuronal membrane, and therefore can be influenced by drugs that block voltage-gated sodium channels like lamotrigine or phenytoin [30,31], whereas drugs that influence synaptic transmission like memantine have little influence [32].

Changes of motor threshold and area may be simply due to changes of local motor excitability and not necessarily linked to "true" plasticity in the underlying networks [33]. In contrast, the shift of the COG of the motor output map must be considered as a marker for organisational changes in the representation of movements within the primary motor cortex, and therefore as a correlate of "true" motor cortex plasticity [33-35]. In previous studies, it has been extensively demonstrated that drugs affecting cortical excitability (including NMDA-antagonists) are not able to induce "true" cortical plasticity on their own, suggesting that cortical excitability changes might constitute a necessary, but not sufficient factor in the induction of cortical plasticity [12,15]. Thus it is not conceivable that memantine, which is also known to affect motor cortex excitability [32], induces a (lateral) directional shift of the COG on its own, and therefore only masks the training induced medial shift of the COG. Such an effect of memantine alone on the COG could additionally be excluded by comparing the baseline maps obtained prior to the motor learning with placebo and memantine in the ascending dosage experiment, which did not significantly differ from each other. Our results therefore strongly suggest that memantine is able to block training-induced motor cortex plasticity.

Similar use-dependent alterations of movement representations were observed in the primary motor cortex of adult squirrel monkeys by Nudo et al. [36]. They discovered that after training there was an area expansion of dual-response representations, i.e., cortical sectors over which stimulation produced movements over two or more joints. This may explain the shift of the APB motor output map towards the representation of the co-contracted deltoid muscle in our study. This representational shift can be referred to a principle presented by Hebb, who suggested that individual neurons could participate in different cell assemblies and be involved in multiple functions and representations [37]. The synchronisation or pairing of impulses would then lead to an increase of the excitability of specific neuronal populations, and to a strengthening of the efficacy of their synaptic pathways [10]. This strengthening of synaptic efficacy would involve LTP-like mechanisms, and require the activation of NMDA receptors [21,22]. The fact that the NMDA antagonist memantine was able to prevent cortical plasticity supports this view and confirms previous findings [11,15].

A significant effect of memantine on motor cortex plasticity could be observed in the ascending dosage experiment, whereas the single dosage experiment failed to show a significant difference between memantine and placebo. Since memantine serum levels were markedly higher in this group, we cannot completely rule out that the differential effects of the two administration regimens are linked to these differences in the serum levels. However, in an additional control experiment, an effective blockade of training-induced cortical plasticity was also reached by a repeated administration of a low memantine dosage, with memantine serum levels comparable to those measured after administration of a higher single dosage. Besides, correlation analysis failed to detect a linear correlation between memantine serum levels and COG shifts in individual subjects. These additional findings suggest that the repeated administration of memantine over 8 days was more important for the blocking effect than the memantine serum levels. In previous studies, it has been demonstrated that after subchronic NMDA receptor blockade over seven days, regulatory mechanisms occur at a cellular level, including a down-regulation of cortical NMDA receptors due to a reduction of the glutamate binding site [38,39]. Such mechanisms might also have occurred in our study after 8 days of memantine administration in both the ascending dosage experiment and the control experiment using a repeated low dosage, and therefore have contributed to the effective blockade of training-induced cortical plasticity regardless of memantine serum levels.

During one hour of training, a significant improvement was observed in motor performance, which was not affected by memantine administration, neither in the single dosage nor in the ascending dosage experiment. There was also no significant correlation between the performance improvement and the changes of one of the mapping parameters. This lack of correlation also corresponds to the results reported in previous studies [10,12], which did not reveal a correlation between the improvement of motor performance and the shift of the motor output map. It supports the view that the repetition of a synchronised movement is more important for the induction of cortical plasticity than the improvement of the motor performance.

## Conclusion

Previous clinical studies, which used the NMDA antagonist memantine to treat patients e.g. with chronic phantom limb pain, failed to prove any therapeutic effect of memantine in these patients [40-42]. This might be due to the fact that pain-related reorganisation in these chronic patients was already fixed and based on structural changes, hence no longer dependent on NMDA receptor activation. However, from a theoretical point of view, preventing cortical plasticity by blocking NMDA receptors might be a promising clinical approach for the prevention of phantom limb pain if the NMDA antagonist is administered before amputation. The present study suggests that memantine is a drug that could be effective for this purpose, and should be evaluated in further clinical studies. It also emphasizes the importance of an adequate dosage regime, since it would be probably ineffective if administered as a single dosage. Repeated administration seems to be more important than the reached serum levels, possibly allowing the use of lower dosages which are better tolerated.

## Methods

### Subjects and study design

We investigated 20 healthy right-handed subjects (12 men and eight women, aged 19 – 41 years, mean age 27.7 ± 6.8 years), who were all unrelated to the medical field. They all gave their written informed consent, and the protocol was approved by the local ethical committee. All subjects participated either in the single dosage experiment, or in the ascending dosage experiment, or in the control experiment with a repeated low dosage.

Single dosage experiment: Eight subjects (four men and four women aged between 20 and 34 years) had to participate in two experimental sessions using a randomised double-blinded cross-over study design, with both the researcher and the participating subject being blinded to the experimental condition. They were randomly split into two groups. The first group, made up of four subjects, started with a session including the administration of a single dose of memantine (20 mg to 35 mg, adapted to the individual body weight), the other group with placebo administration. This process was reversed after an interval of at least 14 days. Each session started with a TMS mapping of the right abductor pollicis brevis (APB) muscle as described below. The drug was administered immediately after finishing this mapping procedure, and blood samples were drawn every hour afterwards in order to monitor memantine serum levels. Four hours after drug intake, subjects had to perform a repetitive motor task as described below. Immediately after this motor training, a second TMS mapping of the right APB muscle was performed in order to assess training-induced changes of the APB motor output map.

Ascending dosage experiment: Again a randomised double-blinded cross-over study design was used to study seven other subjects (four men and three women) in this experiment, with the researcher and the participating subject being blinded to the experimental condition. Subjects were randomly split into 2 groups, one group receiving placebo in a first and memantine in a second session, and one group receiving memantine in a first and placebo in a second session, each over a period of 8 days. There was a wash out phase of at least 14 days between both sessions in each subject. Memantine was initially given with a dosage of 10 mg daily, and augmented in steps of 10 mg every 3 days up to 30 mg daily. At day 8, memantine serum levels were determined, and a TMS mapping of the APB muscle was performed twice, immediately before and after one hour of repetitive motor training. Mapping procedures and motor training were identical as in experiment 1.

Control experiment with a repeated low dosage: Five subjects (four men and one woman) participated in this control experiment, receiving a daily dosage of 10 mg memantine over a period of 8 days. In this control experiment, there was no placebo condition, and the researcher as well as the subject was aware of the administered drug. At day 8, memantine serum levels were determined, and a TMS mapping of the APB muscle was performed twice, immediately before and after one hour of repetitive motor training. Mapping procedures and motor training were identical as in the other experiments. This experiment was designed to achieve memantine serum levels comparable to the single dosage experiment, in order to determine whether the memantine serum levels or the repeated administration might be more important for the drug's effect on training- induced cortical plasticity.

### Memantine serum level

Immediately after each blood withdrawal, the sample was centrifuged, and the serum was frozen at -70°C. After finishing the study, all frozen samples were shipped together to the Department of Pharmacological Research, Merz Pharmaceuticals, Frankfurt am Main, Germany, where memantine serum levels were assayed with a gas-chromatographic system coupled to a mass selective detector (for details, see [43-45]). Results were expressed as ng/ml concentrations of memantine free base, and not as concentrations of memantine hydrochloride.

### Transcranial magnetic stimulation (TMS) mapping

TMS was performed with a Magstim 200 HP device (The Magstim Company) and a figure-of-eight shaped coil (outside diameter 8.7 cm, peak magnetic field strength 2.2T, peak electric field strength 660 V/m), which predominantly stimulates neural structures under its centre. During the whole mapping procedure the coil was held tangentially to the head in an anterior-posterior direction, with the grip pointing backwards. Motor evoked potentials (MEP) were recorded with surface electrodes from the right abductor pollicis brevis muscle (APB) and stored on an EMG device (Neuropack 8, Nihon Kohden). The band pass was 20 Hz to 2 kHz, the gain 0.1 to 1 mV/D. The magnetic stimuli were delivered while the subjects were seated comfortably in a chair. During the whole examination, muscle relaxation was monitored with surface electrodes by EMG (gain 0.1 mV/D). Motor threshold (MT) was determined at rest to the nearest 1% of the stimulator output, and was defined as the minimum intensity which produced five motor evoked potentials >50 μV out of ten trials [46]. Threshold was determined over the scalp position were TMS previously elicited the highest amplitude. Starting at this scalp position and using a stimulation intensity of 110 % of the motor threshold, the motor cortex was examined systematically in rostral, dorsal, medial and lateral directions in steps of 1 cm until no further MEP could be elicited. The positions were identified with the help of a tight fitting cap with a coordinate system drawn on it (1 × 1 cm width). Cz was identified as the intersection of the interaural line and the connection between nasion and inion, which made it possible to localize the coordinates relative to Cz. The x-coordinate was used to indicate the distance in anterior-posterior direction relative to Cz, and the y-coordinate to indicate the distance in medio-lateral direction. Coordinates of Cz were defined as 0/0. Eight stimuli were applied to each position of the grid, and the averaged peak-to-peak MEP amplitude was considered for further statistical analysis. Averaged amplitudes smaller than 10 μV were regarded as zero. Afterwards, we calculated the sum of all MEP amplitudes of the motor output map (SOA), and its amplitude-weighted centre of gravity (COG). The x and y-coordinates of the centre of gravity (COG) are derived from the distribution of MEP amplitudes within the motor output area. They were calculated according to the formula [Σ(x*z)/Σz] or [Σ(y*z)/Σz], where x or y is the position along the x or y axis, and z is the amplitude at this position [10].

As additional mapping parameters were assessed:

- the number of active stimulation sites, i.e. the number of scalp sites from which MEPs could be elicited was used as a marker for the area size of the motor output map, each position equaling 1 cm^2^,

- the number of "hotspots" of the motor output map, i.e., the number of scalp sites on the coordinate system where TMS elicited MEPs with a mean MEP amplitude of >150 μV, and

- the mean MEP amplitude across all stimulation sites, which was calculated by dividing the SOA by the number of active stimulation sites.

### Motor training

In each experimental session, the subjects had to perform a repetitive motor task. This motor task consisted of a synchronised contraction of the deltoid and abductor pollicis brevis (APB) muscle. The participants were instructed to make brisk and short movements of both muscles as synchronously as possible. Approximately three co-contractions per minute had to be performed over one hour. After each single co-contraction, the latency difference between the onsets of muscle contractions was determined using EMG-monitoring with surface electrodes on both muscles. These latency differences of voluntary EMG-activity allowed us to evaluate motor performance. The subjects were informed about the results of their performance and encouraged to improve it [11].

### Statistical analysis

Student's paired t-test served to assess intraindividual changes in mapping parameters before and after motor training without memantine intake, to evaluate intrasubject reproducibility of the neurophysiologic data by comparing the maps of individual subjects obtained prior to motor learning in different sessions, and to examine the effect of memantine alone by comparing the baseline maps between the memantine and the placebo condition in the ascending dosage experiment. Student's paired t-test was also used in the control experiment to assess changes in mapping parameters before and after motor training after repeated administration of a low dosage of memantine. To evaluate differences of the TMS mapping parameters between placebo and memantine sessions we used an ANOVA for repeated measurements (main factors "training", i.e., pre vs. post, and "drug" i.e., memantine vs. placebo). The Greenhouse-Geisser procedure was used with epsilon-corrected degrees of freedom, where data showed significant deviations from sphericity. To evaluate the effect of repetitive co-contraction on motor performance, the mean latency differences between the onsets of both muscles for the intervals 0–10 min, 10–20 min, 20–30 min, 30–40 min, 40–50 min and 50–60 min were calculated in each subject and for each session. ANOVA for repeated measurements (main factors: "training duration" and "drug") was performed. Pearson's correlation coefficient r was calculated in order to detect a possible relationship between the pre-post differences of the mapping parameters, memantine serum levels and the improvement of motor task performance, defined as the difference between the mean onset latency difference in the 0–10 min and the 50–60 min interval. For this correlation analysis, only the data obtained after memantine intake were used. For all tests, significance was assumed at the 5 % level.

## Authors' contributions

PS and KW conducted the TMS mapping experiments, performed the statistical analysis and drafted the manuscript. BP, JPM and MT participated in the design and coordination of the study, and in the discussion of the results. All authors read and approved the final manuscript.

## Figures and Tables

**Table 1:** TMS mapping results. Mean ± standard deviation for the different TMS parameters assessed before and after one hour of synchronized movements of the right thumb and shoulder under different conditions

	Single dosage (n= 8)			
	Placebo		Memantine	

	Before training	After training	Before training	After training

x coordinate COG (cm)	1.58 ± 0.61	1.59 ± 0.67	1.43 ± 0.72	1.58 ± 0.62
y coordinate COG (cm)	-4.82 ± 0.44	-4.74 ± 0.41	-4.98 ± 0.56	-4.97 ± 0.65
Motor threshold (%)	34.6 ± 3.0	34.1 ± 2.7	35.0 ± 1.7	34.4 ± 1.9
Area (cm^2^)	14.6 ± 4.4	13.8 ± 2.8	15.5 ± 5.2	16.1 ± 2.8
Sum of amplitudes (μV)	1584.6 ± 999.6	1412.0 ± 797.8	2022.4 ± 1193.6	1733.0 ± 756.6
Number of "hotspots"	3.4 ± 2.4	2.7 ± 2.6	4.2 ± 2.8	3.4 ± 2.3
Mean amplitude (μV)	116.1 ± 94.9	100.4 ± 49.9	161.9 ± 180.6	111.4 ± 53.9

	Ascending dosage over 8 days (n = 7)			
				

	Placebo		Memantine	

	Before training	After training	Before training	After training

x coordinate COG (cm)	1.21 ± 0.60	1.38 ± 0.59	1.49 ± 0.64	1.53 ± 0.66
y coordinate COG (cm)	-5.46 ± 0.69	-5.34 ± 0.61	-5.36 ± 0.50	-5.44 ± 0.59
Motor threshold (%)	40.6 ± 7.1	39.7 ± 6.5	41.3 ± 7.3	40.9 ± 7.0
Area (cm^2^)	16.6 ± 3.8	15.7 ± 3.6	15.9 ± 3.6	16.3 ± 3.7
Sum of amplitudes (μV)	1901.6 ± 1515.8	1535.1 ± 939.3	1744.4 ± 1086.0	1726.7 ± 808.6
Number of "hotspots"	4.4 ± 3.5	3.6 ± 2.6	3.9 ± 3.1	3.6 ± 2.8
Mean amplitude (μV)	120.5 ± 95.4	104.3 ± 65.0	107.2 ± 52.7	107.6 ± 45.2
				

	Low dosage (10 mg/d) over 8 days (n = 5)			

			Memantine	

			Before training	After training

x coordinate COG (cm)			0.80 ± 0.46	0.62 ± 0.71
y coordinate COG (cm)			-5.12 ± 0.46	-5.20 ± 0.53
Motor threshold (%)			38.4 ± 8.3	38.8 ± 12.1
Area (cm^2^)			12.0 ± 4.1	14.6 ± 4.0
Sum of amplitudes (μV)			1852.2 ± 2169.4	1984.6 ± 1686.1
Number of "hotspots"			3.8 ± 3.8	4.0 ± 4.3
Mean amplitude (μV)			131.2 ± 106.1	122.0 ± 92.5
